# Lipolysis stimulating peptides of potato protein hydrolysate effectively suppresses high-fat-diet-induced hepatocyte apoptosis and fibrosis in aging rats

**DOI:** 10.3402/fnr.v60.31417

**Published:** 2016-07-12

**Authors:** Wen-Dee Chiang, Chih Yang Huang, Catherine Reena Paul, Zong-Yan Lee, Wan-Teng Lin

**Affiliations:** 1 Department of Food Science, College of Agriculture, Tunghai University, Taichung, Taiwan; 2 Graduate Institute of Basic Medical Science, China Medical University, Taichung, Taiwan; 3 School of Chinese Medicine, China Medical University, Taichung, Taiwan; 4 Department of Health and Nutrition Biotechnology, Asia University, Taichung, Taiwan; 5 Department of Hospitality Management, College of Agriculture, Tunghai University, Taichung, Taiwan

**Keywords:** apoptosis, fibrosis, high fat diet, lipolysis stimulating protein hydrolysates, obesity, APPH

## Abstract

**Background:**

Non-alcoholic fatty liver disease (NAFLD) is one of the most common outcomes of obesity and is characterized by the accumulation of triglycerides, increased tissue apoptosis, and fibrosis. NAFLD is more common among elderly than in younger age groups, and it causes serious hepatic complications.

**Objective:**

In this study, alcalase treatment derived potato protein hydrolysate (APPH) with lipolysis-stimulating property has been evaluated for its efficiency to provide hepato-protection in a high-fat-diet (HFD)-fed aging rats.

**Design:**

Twenty-four-month-old SD rats were randomly divided into six groups (*n*=8): aged rats fed with standard chow, HFD-induced aged obese rats, HFD with low-dose (15 mg/kg/day) APPH treatment, HFD with moderate (45 mg/kg/day) APPH treatment, HFD with high (75 mg/kg/day) APPH treatment, and HFD with probucol.

**Results:**

APPH was found to reduce the NAFLD-related effects in rat livers induced by HFD and all of the HFD-fed rats exhibited heavier body weight than those with control chow diet. However, the HFD-induced hepatic fat accumulation was effectively attenuated in rats administered with low (15 mg/kg/day), moderate (45 mg/kg/day), and high (75 mg/kg/day) doses of APPH. APPH oral administration also suppressed the hepatic apoptosis- and fibrosis-related proteins induced by HFD.

**Conclusions:**

Our results thus indicate that APPH potentially attenuates hepatic lipid accumulation and anti-apoptosis and fibrosis effects in HFD-induced rats. APPH may have therapeutic potential in the amelioration of NAFLD liver damage.

Aging is a biological process characterized by a progressive decrease in physiological capacity and increases in age-related diseases, and the process of aging is frequently associated with progressive insulin resistance and associated decline in blood sugar regulation, arising from deteriorated control of glucose production and triglyceride secretion in the liver (1). Aging therefore increases the occurrence of metabolic disorders such as dyslipidemia and hyperinsulinemia (2–4). Non-alcoholic fatty liver disease (NAFLD) is a common metabolic syndrome that progresses into chronic liver disease in obese adults (5, 6). Hypernutrition or insulin resistance potentially causes imbalance in the uptake and removal of lipids in the liver, leading to excess accumulation of hepatic triglycerides (7). NAFLD occurs more often in the middle-aged and in the elderly as the risk factors for NAFLD increases with aging (8). Hepatic apoptosis is one of the reasons for NAFLD-induced liver injury (9, 10). A higher incidence of apoptosis also leads to hepatic fibrosis. Therefore, inhibition of hepatic apoptosis is a potential approach in treating NAFLD. As there are no drugs so far that effectively treat NAFLD, diet control and diet-related treatment become more crucial in preventing NAFLD (7).

Potato (*Solanum tuberosum* L.) is an important food crop and a major source of high-quality proteins, minerals, and antioxidants, including vitamin C (11). Recent reports highlight the efficiency of the potato as an effective functional food with various health-promoting benefits such as protection from degenerative diseases, cancer, heart disease, and hypertension (11). Enzymatic hydrolysis of dietary proteins produces potential bioactive peptides with altered molecular size, hydrophobicity, and polar groups of peptides (12). Alcalase treatment–derived potato protein hydrolysate (APPH) with lipolysis-stimulating property was found to have the potential to act as an efficient anti-obesity diet ingredient (13–15). Recent in vitro studies on the stability of APPH against gastric proteases also show that APPH is highly resistant to proteolytic digestion (15).

In this study, we investigated the effect of APPH on fatty liver–associated hepatic apoptosis and fibrosis in aged obese rats. Twenty-four-month-old male SD rats that were fed with HFD for 60 days (HFD group) were found to develop symptoms of NAFLD with hepatic apoptosis and fibrosis. However, HFD-fed rats treated with different doses of APPH for the final 30 days showed a significant reduction in HFD-related apoptosis and fibrosis. The administration of APPH also protected the liver by reducing the accumulation of lipid droplets and regulating the level of apoptosis- and fibrosis-related events. APPH administration can therefore be considered as a potential therapeutic agent to ameliorate aging-associated NAFLD effects.

## Materials and methods

### APPH preparation

**Fig. 1:**
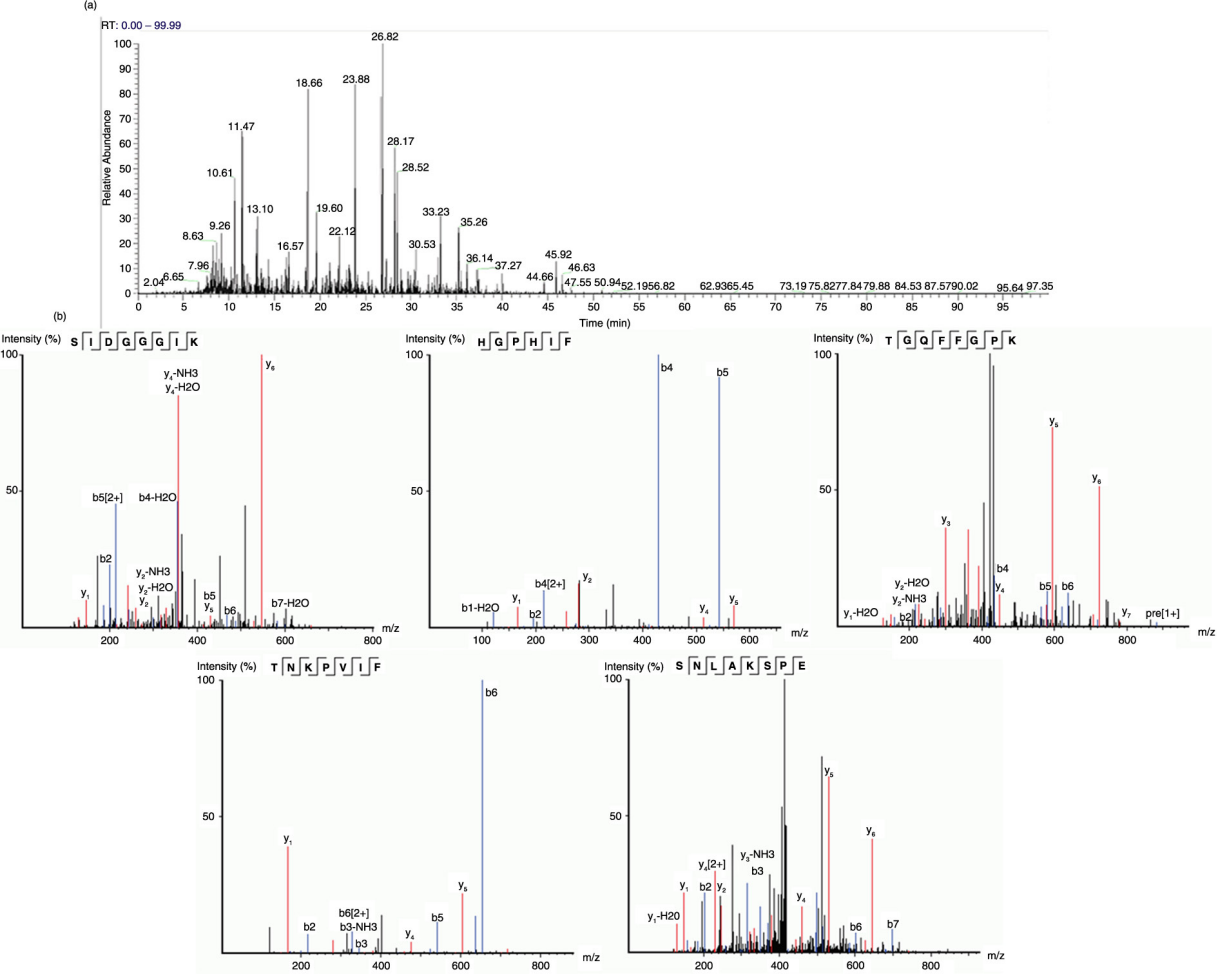
Characterization of lipolysis stimulating APPH. (a) MS/MS/TIC chromatogram for APPH. (b) Ion series for chosen peptides from the MS/MS/TIC chromatogram showing sequence homogeneity with patatin.

APPH was prepared, purified, and characterized as reported previously; the characteristics were consistent with the composition of APPH mentioned in earlier reports (13). Briefly, a reaction mixture containing 2.5% commercially purchased potato protein (Han-Sient Corporation, Taipei, Taiwan) and 1% alcalase enzyme (Nono Nodisk A/S, Bagsvaerd, Denmark) were used to produce potato protein hydrolysate (~81% protein) by subjecting it to alcalase enzyme hydrolysis for 2 h. The molecular size of the resultant APPH was determined using different membrane cut-off filters. It was confirmed, as in the previous report, that 5% of the content was >6,000 Da, 40% was between 1,000 and 6,000 Da, and 55% was <1,000 Da size. The hydrolysate was vacuum-dried and stored for future use. The degree of hydrolysis of the product was more than 9.5% and the hydrolysate was fractionated by reverse phase HPLC; characterized according to the methods reported previously (13, 16) by MS/MS/TIC and by analyzing the data with TurboSequest (Thermo Fisher Scientific, MA, USA) against protein database (UniProt, *Solanum tuberosum*) and the results revealed the presence of peptides with patatin sequences (Fig. 1).

### Animal experiments

This study was conducted according to the IACUC-100-12 protocol after appropriate ethical approval from the Institutional Animal Care and Use Committee (IACUC) of the China Medical University, Taiwan. Five-week-old male SD rats were housed in cages maintained at 24±2°C and 55±10% humidity with a 12-h light cycle, and the rats were allowed to feed on normal laboratory chow for 22 months. The rats were randomly divided into six groups (*n*=8): aged rats fed with standard chow, aged rats fed with HFD, aged rats with HFD and low-dose (15 mg/kg/day) APPH treatment, aged rats with HFD and moderate dose (45 mg/kg/day) APPH treatment, aged rats with HFD and high-dose (75 mg/kg/day) APPH treatment, and aged rats with HFD and probucol. The animals were fed with a standard laboratory diet (PMI Nutrition International, Brentwood, MO, USA) and provided with reverse osmosis–treated water *ad libitum*. In the next 8 weeks, obesity was induced by giving HFD (58Y1, LabDiet, Missouri, USA) containing 60% of energy as fat. The treatment was administrated orally using non-flexible needless feeding gavage simultaneously during the next 30 days of HFD feeding course. The probucol treatment group rats were fed with high fat diet and probucol (500 mg/kg/day).

### Hematoxylin and eosin staining

The livers were excised; soaked in formalin; dehydrated by passing through 100, 95, and 75% alcohol sequentially and then embedded in paraffin wax. The embedded tissue blocks were cut into 0.2-µm-thick sections and de-paraffinized by soaking in xylene. The slices were stained using hematoxylin and eosin (H&E) and rinsed with water. Photomicrographs were obtained using Zeiss Axiophot microscopes (Carl Zeiss Microscopy, Thornwood, NY, USA).

### Terminal deoxynucleotide transferase-mediated dUTP Nick End Labeling (TUNEL)

The tissue sections were incubated with proteinase K, washed in phosphate-buffered saline, incubated with permeabilization solution, blocking buffer, and then washed two times with PBS. The sections were stained with terminal deoxynucleotidyl transferase and fluorescein isothiocyanate- dUTP (Roche Applied Science, Indianapolis, IN, USA) for 60 min at 37°C in order to detect the number of apoptotic nuclei. The tissue sections were also stained with 0.1 µg/mL 4’,6-diamidino-2-phenylindole (DAPI) for 5 min, and the nuclei were detected by UV light microscopy at 454 nm. Photomicrographs were obtained using a Zeiss Axiophot microscope. All counts were performed by at least three independent individuals in a blinded manner.

### Masson's trichrome staining

Liver tissues were fixed in formalin, embedded in paraffin, and sectioned. Slides were hydrated through a series of graded alcohols (100, 95, and 75%), for 15 min each. The slides were then stained with Masson's trichrome dye. And photomicrographs were obtained using the Zeiss Axiophot microscopes.

### Tissue protein extraction and western blotting

Proteins were obtained by homogenizing the liver tissue in lysis buffer (100 mg/mL). The homogenates were placed on ice and then centrifuged at 12,000 *g* for 40 min. The supernatants were collected and stored at −80°C for further experiments. The extract protein concentration was determined using the Lowry protein assay method. Protein samples were separated in a 12% SDS polyacrylamide gel electrophoresis (SDS-PAGE) using 75 V of constant power supply. Proteins were then transferred to PVDF (GE Healthcare Life Sciences, Pittsburgh, PA, USA) membranes using 50 V current for 3 h. The membranes were incubated in 3% bovine serum albumin (BSA) in TBS buffer and the primary antibodies (Santa Cruz Biotechnology, Santa Cruz, CA, USA) were added onto the membranes for conjugation with specific proteins. Horseradish peroxidase–labelled secondary antibodies were used for detection, and pictures were finally taken with Fujifilm LAS-3000 (GE Healthcare Life Sciences).

### Statistical analysis

The results shown are the means±SD of three independent experiments. Statistical analysis was performed by one-way analysis of variants. For paired samples, the Student's *t* test was used.

## Results

### APPH administration alleviated HFD-induced effects

**Fig. 2:**
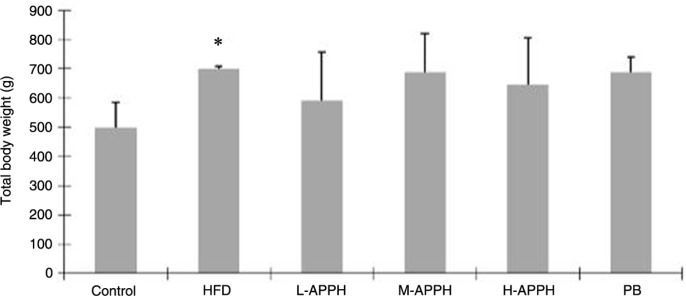
Effect of APPH on total body weight changes. The changes in body weight (g) were measured in the controls and in the treatment groups were determined. The results represent mean±SD (*n*=8). *P<0.05, when compared with control group (one-way ANOVA analysis).

**Fig. 3:**
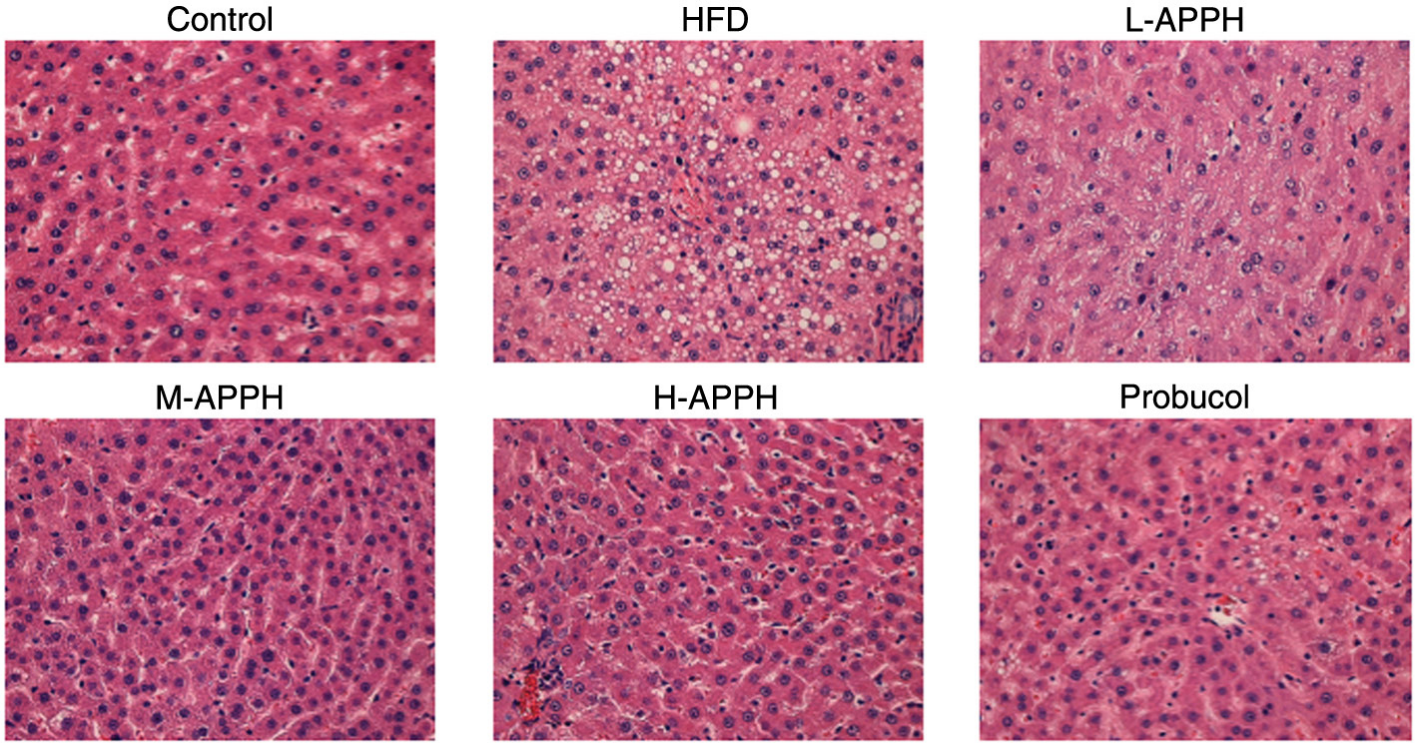
Hematoxylin and eosin (H&E) staining of tissue sections showing the liver tissue architecture and lipid droplets. Histopathological analysis of hepatic sections of control rats, high-fat-diet-fed rats, and the treatment group rats was performed. Magnification 400x.

The HFD intake induced several notable changes in the test rats. The rats fed with HFD showed significant increase in body weight compared to the control rats. Although some reduction in body weight was observed, the effect of APPH and probucol on the HFD induced increase in body weight was not statistically significant (Fig. 2). Massive fat accumulation in the livers of rats fed with HFD was also observed from H&E-stained liver sections (Fig. 3). However, APPH treatment effectively reduced the fat accumulation in the liver tissue. Moderate and high dose of APPH were comparatively more effective in attenuating the fat accumulation than the probucol treatment group.

### APPH administration supresses apoptosis

**Fig. 4:**
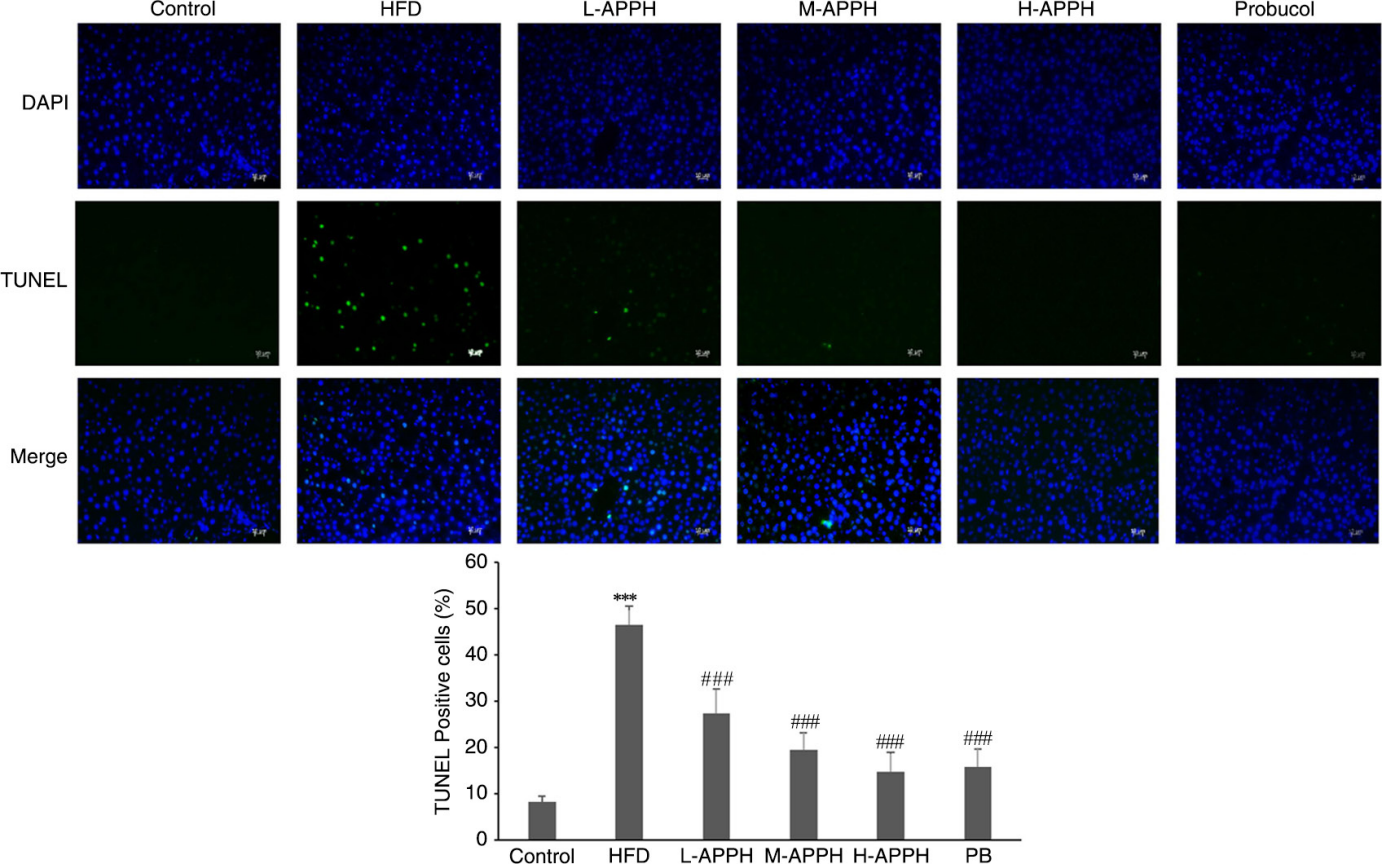
Effect of APPH on cellular apoptosis. DAPI and TUNEL stained liver sections show the degree of apoptosis in control rats, high-fat-diet-fed rats, and the treatment group rats. Magnification 400x. ***P<0.001, when compared with control group and ^###^ *P*<0.001 when compared with HFD group.

TUNEL and DAPI staining of the liver sections showed more number of TUNEL positive cells in HFD rat groups indicating a hepatotoxic effect caused by HFD feeding. However, treatment with low, moderate, and high levels of APPH supressed the number of apoptosis nuclei in a dose-dependent manner (Fig. 4).

### APPH administration regulates apoptosis- and survival-related proteins

**Fig. 5:**
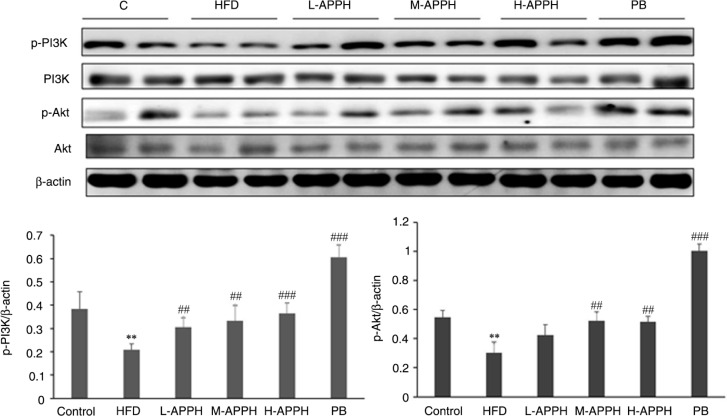
Effect of APPH on survival proteins. The cell survival protein levels such as p-PI3K and p-Akt in the 24-month-old control rats, high-fat-diet-fed rats, and the treatment group rats. **P<0.01 and ***P<0.001, when compared with control group ^#^ *P*<0.05, ^##^ *P*<0.01 and ^###^ *P*<0.001 when compared with the HFD group.

**Fig. 6:**
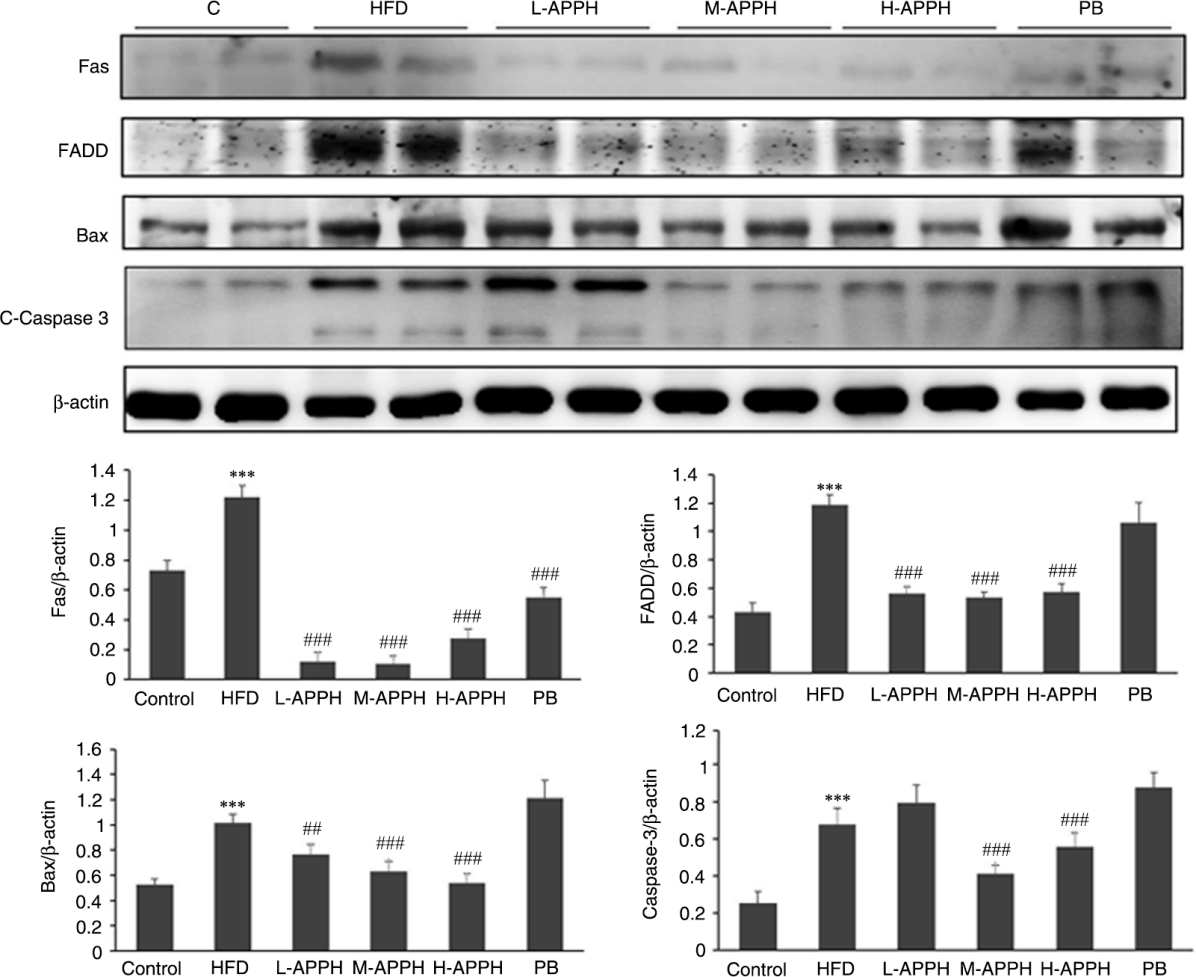
Effect of APPH on apoptosis marker proteins. The levels of apoptosis protein such as Fas, FADD, Bax, and caspase 3 in the 24-month-old control rats, high-fat diet-fed rats, and the treatment group rats. ***P<0.001, when compared with control group, ^##^ *P*<0.01 and ^###^ *P*<0.001 when compared with HFD group.

The protein expression in the HFD-fed rat livers, when analyzed by western blotting, revealed that the incidence of fatty liver in rats is correlated with suppression in the levels of activated phosphatidylinositol 3-kinase (p-PI3K) and serine–threonine kinase (p-Akt) and elevation in pro-apoptotic protein levels such as Fas receptor (Fas), Fas-Associated protein with Death Domain (FADD), Bcl-2 associated X protein (Bax), and activated caspase 3. However, in the rat groups administered with low, moderate, and high levels of APPH, the levels of survival proteins were elevated (Fig. 5) and the levels of apoptosis proteins were suppressed (Fig. 6).

### Effect of APPH on liver fibrosis

**Fig. 7:**
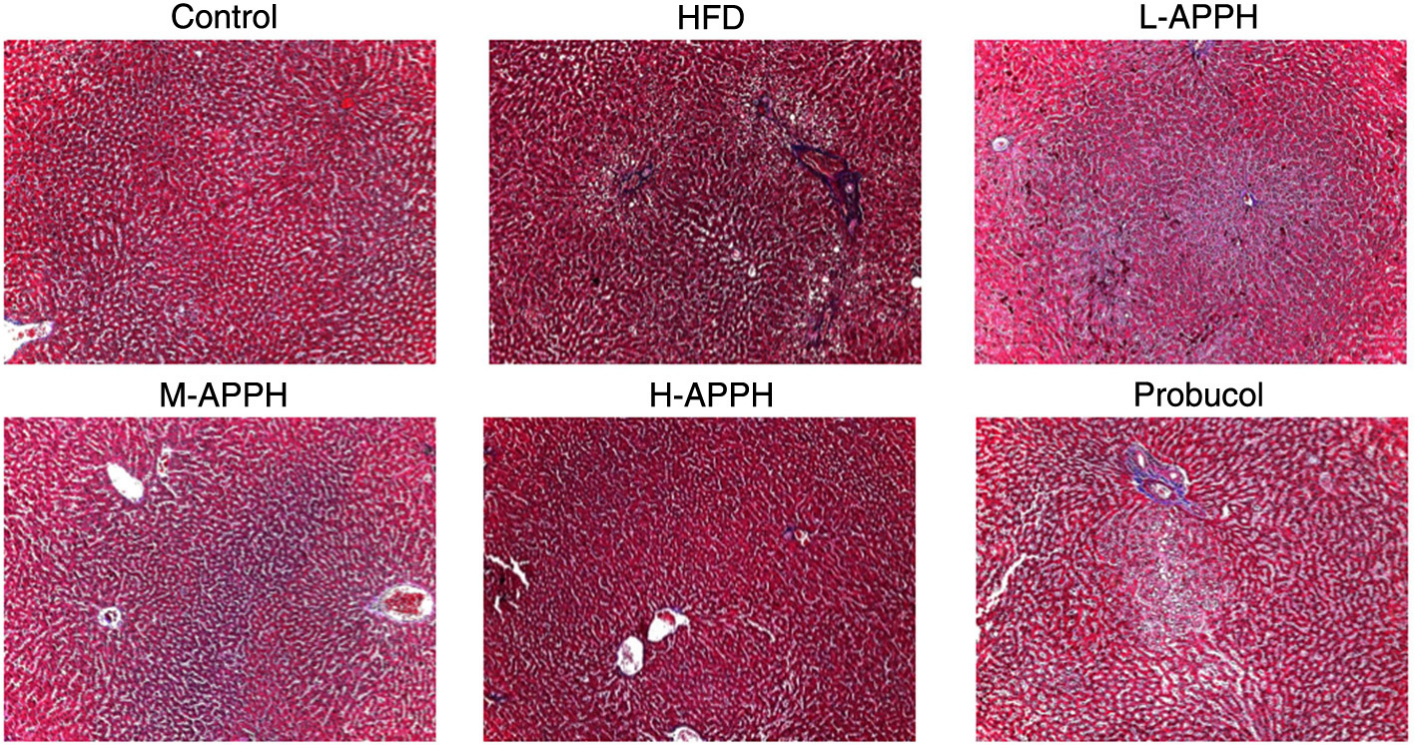
Effect of APPH on fibrosis. Masson's trichrome staining in liver tissue sections from 24-month-old control rats, high-fat-diet-fed rats, and the treatment group rats. Magnification 400x.

Masson's trichrome staining of LV tissue sections revealed that HFD in aging rats induced liver fibrosis (Fig. 7). Tissue sections showed prominent blue staining in the HFD-fed rat liver sections. However, treatment with APPH effectively reduced the fibrosis effects induced by HFD. The results show that APPH has more potential than probucol to inhibit HFD associated liver fibrosis in hamster livers.

## Discussion

Age-related disorders are widespread throughout the world and are the leading cause of death in developed countries. The deterioration of organ structure and function during aging is associated with oxidative stress, genetic instability, and the disruption of homeostatic pathways, resulting in metabolic syndromes such as obesity, insulin resistance, impaired glucose tolerance or overt diabetes, hypertension, dyslipidemia, and cardiovascular complications (16). Diabetes is also recognized to accelerate aging, but the mechanisms linking diabetes and aging are poorly understood (17).

Obesity is a major cause of insulin resistance and is linked to several common diseases such as type two diabetes, cardiovascular disease, NAFLD, and various cancers (18–20). The HFD-fed rats used in our experiments displayed liver apoptosis and fibrosis symptoms as determined from their liver histology. APPH effectively reduced the liver fat accumulation; however, this change would have caused an increase in fat accumulation elsewhere in the form of visceral or the adipose fat. Therefore, changes in the liver lipid mass were not reflected on the total body weight differences post APPH treatment. The potato proteins have been classified tentatively into three classes: patatin family (41 kDa; ≈40% of total soluble protein), protease inhibitors (5–25 kDa; ≈50% of total soluble protein), and others (21). Patatin exhibits a known lipid acyl hydrolase (LAH) activity (21). APPH contains fragments of lipolysis stimulating peptides such as patatin that has proven anti-obesity effect in rodents. APPH administration is known to confer cardiac protection and improve heart function against HFD-induced effects by attenuating apoptosis and by improving cardiomyocyte survival mechanism (13). In aging rats, APPH administration efficiently reduces serum triacylglycerol and total cholesterol (TC) levels and provides cardio protection to the HFD-fed ailing rats (14). Our present study reveals the effect of APPH in preventing HFD-induced fatty liver and liver fibrosis in aging rats.

The causes of chronic liver diseases are known to be multifactorial, involving obesity, insulin resistance, inflammation, and oxidative stress. Targeting these pathways is considered a potential treatment approach but the strategy has either shown limited efficacy or an unfavorable safety profile (22). Increased hepatocyte apoptosis is a prominent feature of liver diseases leading to liver damage, severe inflammation, and fibrosis. Inflammatory cytokines from hepatocytes in response to liver injury may lead to NAFLD (23, 24). Two major cellular pathways are attributed to the induction and progression of apoptosis in response to different types of stimuli (25). The intrinsic pathway involves the disruption of mitochondrial membrane potential and release of cytochrome *c*, thereby triggering caspase 9. The extrinsic pathway involves activation of death receptors such as the Fas receptor, recruitment of the adaptor molecule FADD, and activation of caspase 8 (26). The members of the Bcl-2 family of proteins-Bcl-2 and Bax, that are, respectively, anti-apoptotic and pro-apoptotic factors, are crucial markers of the events in apoptosis (26). The results indicate that the extrinsic apoptosis pathway proteins that were elevated in HFD-fed rats were regulated when treated with APPH. The APPH also up-regulated Bax in the livers of HFD-fed rats. Therefore, our results indicate that HFD increases apoptosis in rat livers but is regulated when treated with APPH.

Obesity is known to be associated with PI3K/AKT pathway deregulation (27). The IGF1R mediated activation of PI3K and AKT prompts immune cell activation by regulating key inflammatory cytokines (28, 29). APPH treatment has also shown to compensatively enhance IGF1R-PI3K-Akt signaling to improve the cardiomyocyte viability in aging rat hearts (14). In this study, APPH administration increased the levels of PI3K and AKT in HFD-fed rats, indicating improvements in the hepatocyte survival condition. Further, as a consequence of apoptosis associated liver injury, hepatic stellate cells are known to migrate to the apoptosis site to engulf apoptotic bodies. In this process, hepatic stellates are activated to promote the deposition of extracellular matrix and scar formation in the liver (30).

Probucol is a potent antioxidative drug that has been used for the prevention and treatment of atherosclerotic cardiovascular diseases due to its lipid-lowering effects. Probucol decreases serum LDL-C by 10–20% and enhances the excretion of cholesterol into bile (31). Probucol has been known to be effective in attenuating biochemical and histological changes associated with NASH. Probucol treatment normalizes the serum aminotransferase levels and improves hepatic inflammation and fibrosis (32, 33). However, probucol also reduces serum HDL-cholesterol, thereby restricting its therapeutic usage (34). The APPH has shown superior activity compared to probucol and therefore APPH is potentially a valuable alternative to probucol.

In this study, the effect of APPH to attenuate HFD-related apoptosis and fibrosis was determined in HFD-fed aging rat livers. HFD induced changes in the rat bodyweight and expression levels of cellular apoptosis and fibrosis proteins; however, the modulations were found to be regulated when treated with APPH. Therefore, low, moderate, and high concentrations of APPH can potentially rescue rat livers from the effects of HFD.
